# Precise determination of surface band bending in Ga-polar n-GaN films by angular dependent X-Ray photoemission spectroscopy

**DOI:** 10.1038/s41598-019-53236-9

**Published:** 2019-11-18

**Authors:** Yanfei Zhao, Hongwei Gao, Rong Huang, Zengli Huang, Fangsen Li, Jiagui Feng, Qian Sun, An Dingsun, Hui Yang

**Affiliations:** 1 Vacuum Interconnected Nanotech Workstation (Nano-X), Suzhou Institute of Nano-Tech and Nano-Bionics (SINANO), Chinese Academy of Sciences (CAS), Suzhou, 215123 China; 2 Key Laboratory of Nanodevices and Applications, Chinese Academy of Sciences (CAS), Suzhou, 215123 China

**Keywords:** Applied physics, Electronics, photonics and device physics

## Abstract

We present a systematic study of surface band bending in Ga-polar n-GaN with different Si doping concentrations by angular dependent X-ray photoelectron spectroscopy (ADXPS). The binding energies of Ga 3d and N 1 s core levels in n-GaN films increase with increasing the emission angle, i. e., the probing depth, suggesting an upward surface band bending. By fitting the Ga 3d core level spectra at different emission angles and considering the integrated effect of electrostatic potential, the core level energy at the topmost surface layer is well corrected, therefore, the surface band bending is precisely evaluated. For moderately doped GaN, the electrostatic potential can be reflected by the simply linear potential approximation. However, for highly doped GaN samples, in which the photoelectron depth is comparable to the width of the space charge region, quadratic depletion approximation was used for the electrostatic potential to better understand the surface band bending effect. Our work improves the knowledge of surface band bending determination by ADXPS and also paves the way for studying the band bending effect in the interface of GaN based heterostructures.

## Introduction

Group III-nitrides and related alloys have received considerable research interests in recent years mainly due to their advantages in high power/high speed device applications^1–4^. Large spontaneous and piezoelectric polarization effects in Gallium Nitride (GaN) induce large band bending in surface layer and also across the interface of the heterojunction^5^. Surface band bending plays an important role in semiconductor devices by modifying the basic electronic properties and efficiency of them^6^. The effective electron surface band bending results from the complex superposition of contributions from localized surface state charges and polarization charges in wurtzite GaN crystal, and directly affects the device performance^7^. It has been reported that heavily Si doped GaN ohmic layers, in lateral contact to two-dimensional electron gas in the GaN channel, could dramatically improve the DC and RF characteristics in GaN-high electron mobility transistors (GaN-HEMT)^8^. However, there have been few reports on studying the surface band bending in GaN with different doping density, especially in highly doped GaN films. Therefore, it is highly desirable to precisely determine the surface band bending in different doped GaN, to reveal the complication in physics of semiconductor device.

Angular dependent X-ray photoelectron spectroscopy (ADXPS) has been used as a surface sensitive method to determine the surface band bending^9–11^. By decreasing the photoelectron emission angle *θ* respected to the sample surface, the surface sensitivity of the photoelectron spectroscopy can be increased since the detection depth of photoelectron reduces by a factor of sin(*θ*)^12^. Thus, the magnitude of surface band bending can be obtained by measuring the change of photoelectron spectra at different emission angles. However, when considering the real situation of a surface with band bending, the collected core level photoelectron peak is actually an integration of photoelectrons coming from several subsurface atomic layers, instead of the topmost surface layer. In the band bending assessment, the magnitude of surface band bending is determined by the difference in core level energies at the topmost atomic layer and the corresponding value in the bulk, while, the measured photoelectron peak without deconvolution function at the topmost surface layer is expected to be shifted due to the integral effect, which may lead an underestimate or overestimate of the band bending extent^13–15^. To improve the accuracy of analysis, we derive the actual photoelectron peak from different detection depth by performing a method of peak deconvolution to eliminate the integrating effect caused by electrostatic potential. After deconvolution function correction, the actual core level binding energy dependence on the detection depth can be obtained, improving the accuracy of the band bending assessment. In this paper, Ga-polar n-GaN samples with different Si doping densities have been studied by using ADXPS. Ga 3d core level spectra were evaluated correctly by considering the band bending due to the electrostatic potential affected by the doping density combined with localized charges on the surface. In the case of moderate Si doping GaN films, the electrostatic potential can be evaluated simply by a linear relationship to the depth by deconvolution correction. While, in heavily Si doped case, due to the steep space charge potential, the quadratic depletion approximation is more reasonable than linear potential approximation in the surface band bending deconvolution calculation. With deconvolution correction, the surface band bending in different doping density GaN films are precisely determined.

## Results and Discussion

**Figure 1:**
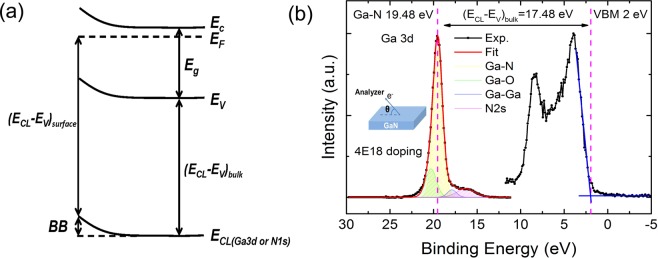
Surface band bending calculation in Ga-polar n-GaN films. (**a**) Schematic surface band bending in Ga-polar n-GaN. (**b**) The measured Ga 3d core level and valance band spectrum of S2 at *θ*= 85*°* are shown by black dots and lines. The Ga 3d spectrum can be fitted into four peaks. The Ga-N, Ga-O, Ga-Ga bonds and N2s peaks are displayed by the yellow, green, blue, magenta lines respectively. The red line represents the fitting envelope. The magenta dashed lines are eye-guides to show the peak position of the Ga-N bond and VBM. The inset shows the definition of emission angle *θ*.

A schematic band diagram for the surface band bending measurement is outlined in Fig. 1(a). The surface band bending (BB) in GaN can be determined from core level binding energy, such as, Ga 3d or N 1 s, and other inherent properties of GaN, as described in the following^6,9^:

1 $${\rm{Band}}\,{\rm{bending}}({\rm{BB}})={({E}_{CL}-{E}_{V})}_{{\rm{bulk}}}+{E}_{g}-{E}_{C}-{({E}_{CL}-{E}_{V})}_{{\rm{surface}}}$$

where, (*E*_*CL*_* − E*_*V*_)_bulk_ is the binding energy difference between core level and valance band maxima in GaN bulk which is a material constant, *E*_*g*_ is the band gap of GaN (3.45 eV^16^), *E*_*C*_ is the position of conduction band with respect to the Fermi level, and depends on doping concentration, (*E*_*CL*_* − E*_*V*_)_surface_ is the core level energy referenced to the Fermi level energy on GaN surface, which would change with the doping density and the surface states. For comparison, three kinds of Si doped GaN films were studied, with doping level of 9 × 10^17^ cm^−3^ (sample 1), 4 × 10^18^ cm^−3^ (sample 2) and 1.4 × 10^19^ cm^−3^ (sample 3), respectively. The doping densities are verified by secondary ion mass spectroscopy (SIMS) measurements. For sample 1 (S1), *E*_c_ is calculated to be 0.03 eV above the Fermi level. When the doping density increases, the Fermi level moves to a higher level. *E*_c_ is calculated to be 0.03 eV and 0.1 eV below the Fermi level for sample 2 (S2) and sample 3 (S3).

Figure 1(b) shows the XPS spectra of the Ga 3d core level spectra, as well as the valance band of S2, collected at emission angle of *θ*= 85° and represented as black dots and lines. By taking Shirley background subtraction and a combination of Gaussian and Lorentzian line shapes, the Ga 3d spectrum can be fitted into four peaks, corresponding to the Ga-N, Ga-O, Ga-Ga bonds and N2s. The fitted highest peak at 19.48 eV originates from Ga-N bond. The valance band maximum (VBM) is determined by the intercept of the slope at the leading edge of the valance band spectrum with the base line, shown by the blue solid line in Fig. 1(b). The energy difference of (*E*_*CL*_* − E*_*V*_)_bulk_ is calculated to be 17.48 eV, which is consistent with the values reported for bulk GaN^17–20^.

**Figure 2:**
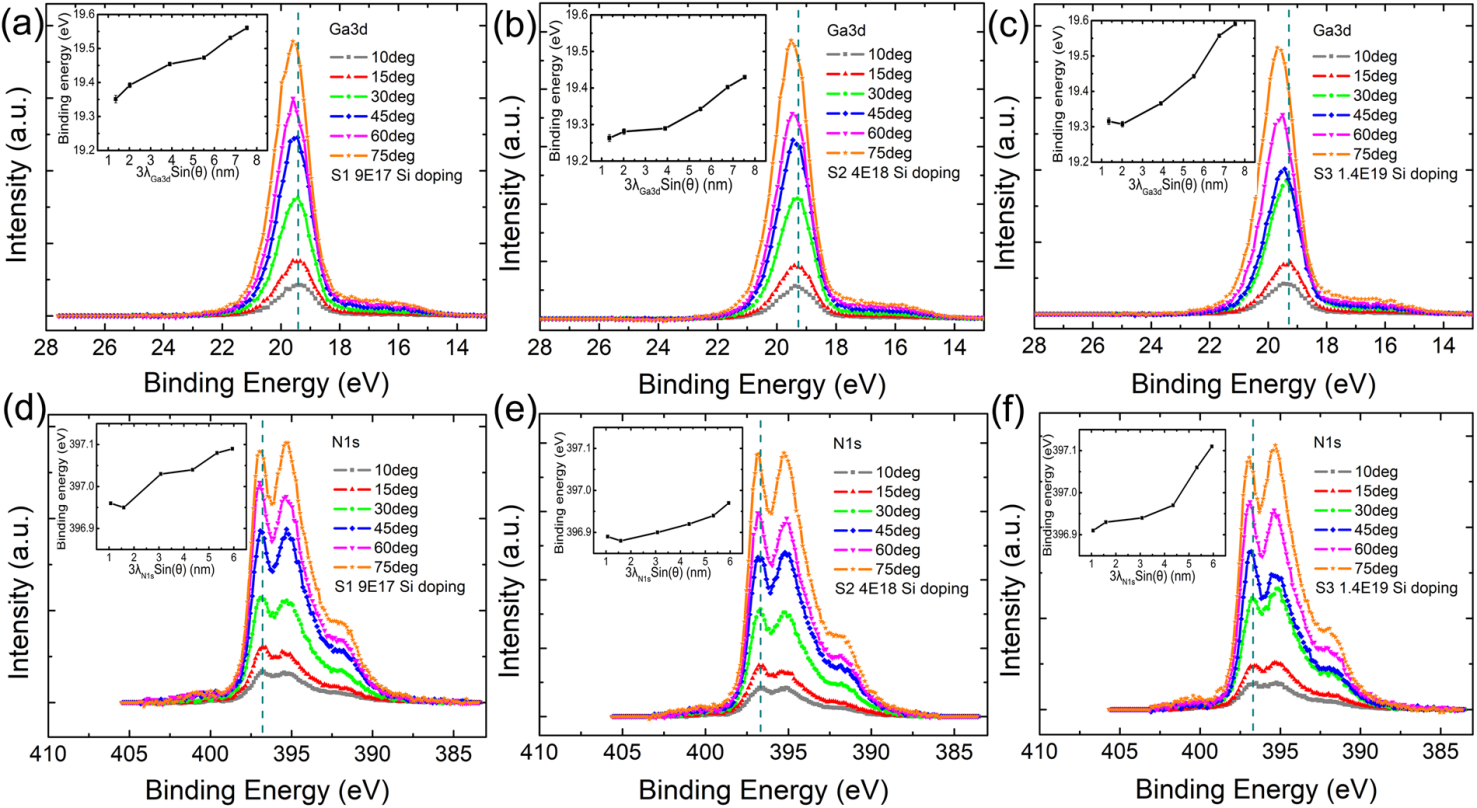
Measured core level spectra of different Si doped GaN samples. (**a–c**) Ga 3d core level spectra measured at different emission angle *θ* for S1, S2 and S3. The inset displays the binding energy of Ga 3d_(Ga-N)_ peak as a function of detection depth. (**d–f**) N 1 s core level spectra measured at different detection angle *θ* for S1, S2 and S3, respectively. The inset displays the binding energy of N 1 s_(N-Ga)_ peak as a function of detection depth. The green dashed lines are eye-guides to highlight the variation of peak position.

Figure 2(a–c) show the Ga 3d core level spectra at different emission angles in different Si doped GaN samples. As displayed in Fig. 2(a), the characteristic Ga 3d_(Ga-N)_ peak in S1 shifts toward higher energy with increasing the emission angle *θ*. Actually, the photoelectron escape depth depends on the emission angle (*θ*) in a simple relation of *λ* sin(*θ*), where *λ* is the inelastic mean free path of photoelectrons. The value of *λ* is 2.6 nm for photoelectrons of Ga 3d in GaN, as calculated by TPP-2M method^21^ in NIST’s database^22^. Thus, the binding energy of Ga 3d_(Ga-N)_ peak increases monotonically with the increase of detection depth (about 3*λ*), as shown in the insert of Fig. 2, implying a sharp upward band bending exists in the GaN surface layer. The similar upward band bending phenomena can also be clearly observed in the Ga 3d _(Ga-N)_ peaks in S2 and S3, as shown in Fig. 2(b,c), respectively. Moreover, the peak shift extent of the highly doped sample (S3) is larger than that of S1 and S2, indicating a larger equivalent internal electric filed exists in S3. It is noted that similar binding energy shift can be also observed in the spectra of N 1 s peaks of different Si doped GaN samples, as shown in Fig. 2(d–f).

**Figure 3:**
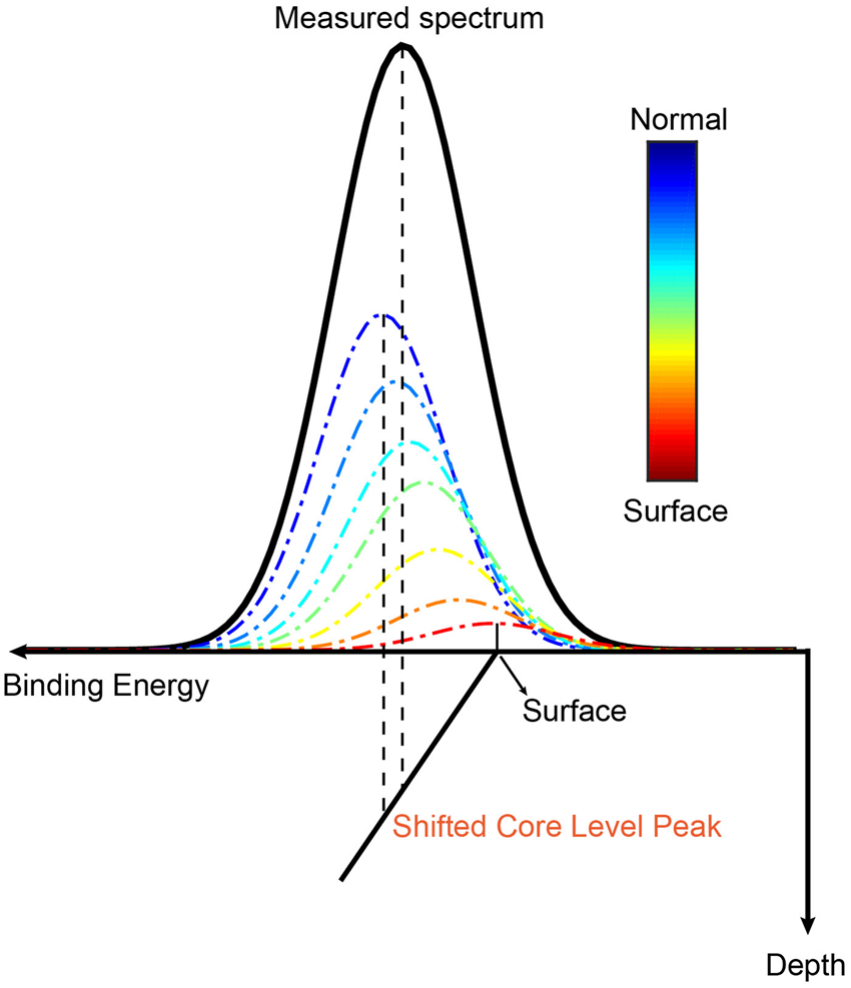
Schematic explaining the deconvolution of the spectra by ADXPS. The shifted core level peak refers to the discrepancy between the measured and actual peak energy position due to the effect of band bending.

As reported previously^5^, an internal potential gradient exists in GaN material due to its large spontaneous polarization. The spontaneous polarization of Ga-polar n-GaN would lead to negative surface bound charges, while the positive donor charges formed in n-GaN compensate the polarization-induced negative surface charge and form an electron accumulation layer, which induces an upward band bending^6^. As shown in Fig. 1(a), the core level (E_CL_), the VBM (E_v_) and the conduction band minimum (E_c_) all bent upward in the Ga-polar n-GaN surface layer. It is known that a core level spectrum obtained by ADXPS is an integration of photoelectrons emitter within the detection depth of the topmost surface. As schematically outlined in Fig. 3, the measured spectrum without deconvolution shown by the solid black line is an integration of the actual spectrum at each depth point along the bend core level displayed by the dash dot color lines. Namely, the dash dot color lines performed the deconvoluted spectra at different depth point. Due to the exponential decay of the XPS intensity, it is educible that the contribution of photoelectrons emitted from a deeper layer is overwhelmed by the contribution of photoelectrons from a shallower layer, leading the peak energy position shifts away from the original binding energy due to the effect of integration. In other words, for an electronic band structure with upward bending, the measured maximum of the integrated photoelectron peak without deconvolution is expected to be shifted away from the deconvoluted binding energy at that depth, guided by the dashed black lines in Fig. 3, and thus gives an under- or overestimation on the band bending magnitude from the values on the very surface.

**Figure 4:**
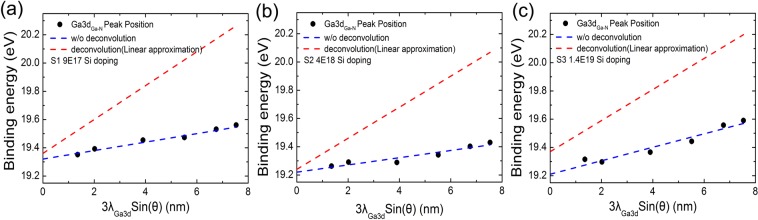
The binding energy of Ga 3d _(Ga-N)_ peak as a function of detection depth with carrier concentration of (**a**) 9 × 10^17^ cm^−3^ (S1), (**b**) 4 × 10^18^ cm^−3^ (S2) and (**c**) 1.4 × 10^19^ cm^−3^ (S3). The blue dashed lines are eye-guides to show the linear fitting of the measured binding energy. The red dashed lines exhibit the actual core level binding energy as a function of detection depth after considering the effect of linear electrostatic potential by Eqs (2, 3).

Based on the ADXPS core level peaks collected at different emission angles, we derive the dependence of photoelectron binding energy on depth in the undersurface layer by taking a method of peak deconvolution for eliminating the integrated effect caused by electrostatic potential. Briefly, a measured core level spectrum as a function of the binding energy, is given as follows^23^:

2 $$I(E)={\int }_{0}^{\infty }{I}_{0}(E-\psi (z))\exp (-\frac{z}{\lambda \,\sin \,\theta })dz$$

where, *z* is the depth from the surface of the bulk, *I*_0_(*E*) refers to the typical core level spectrum with a peak energy of *E*. *ψ*(*z*) stands for the assumed electrostatic potential. *λ* is the inelastic mean free path of photoelectrons. Here, *I*_0_(*E* − *ψ*(z)) can be expressed by the pseudo-Voigt function^24^:

3 $${I}_{0}(E-\psi (z))={I}_{00}[\alpha \,\exp (-\mathrm{ln}\,2\frac{{(E-\psi (z))}^{2}}{{(F/2)}^{2}})+(1-\alpha )\frac{1}{1+\frac{{(E-\psi (z))}^{2}}{{(F/2)}^{2}}}]$$

where, *I*_00_, *α*, and *F* are the core level spectrum intensity, the ratio of Gaussian function, and the full width at half maximum (FWHM) of the core level spectrum, respectively. As shown in Fig. 4, the measured binding energies of Ga 3d _(Ga-N)_ peak are plotted by black solid circles as a function of detection depth. The blue dashed lines are eye-guides to show the peak positions of the binding energies that shift with the detection depth in a linear way, indicating the surface band bending existing in all three samples. However, the peak position of each spectrum collected at different emission angles actually represent the integrated contribution of photoelectrons from different detection depth to sample surface. To figure out the actual binding energy of photoelectron emitted from a certain depth, it is necessary to deintegrate the measured peak according to Eqs (2, 3). First, we consider a uniform internal potential gradient on the surface of Ga-polar n-GaN and ψ(z) is a linear relationship to z. By deconvolution of the spectra measured at different emission angles, we obtained the dependence of the actual core level binding energy on the detection depth, shown by the red dashed lines in Fig. 4. The calculated binding energy at surface *E*_0_, is summarized in Table I. For comparison, the binding energy at the topmost surface layer by the linear fitting of the measured data without deconvolution is expressed by *E*_s_.

**Table I:** The values of (*E*_*CL*_* − E*_*V*_)_surface_ without (w/o) deconvolution and deconvolution by linear approximation as well as the quadratic depletion approximation, and band bending of different doping density GaN samples.

	Doping density (cm^−3^)	${{\boldsymbol{(}}{{\bf{E}}}_{{\bf{C}}{\bf{L}}}{\boldsymbol{-}}{{\bf{E}}}_{{\bf{F}}}{\boldsymbol{)}}}_{{\bf{s}}{\bf{u}}{\bf{r}}{\bf{f}}{\bf{a}}{\bf{c}}{\bf{e}}}$			Band bending (eV)
		w/o deconvolution *E*_s_	Linear approximation *E*_0_	Quadratic approximation *E*_0_	
S1	9 × 10^17^	19.32	19.37	/	1.53 (Linear)
S2	4 × 10^18^	19.22	19.24	/	1.72 (Linear)
S3	1.4 × 10^19^	19.21	19.38	19.30	1.73 (Quadratic)

As shown in Table I, by assuming a linear surface potential *ψ*(z) in GaN, the discrepancy between *E*_0_ and *E*_s_ values are small for S1 and S2, which is consistent with the XPS theory that the major photoemission contribution origins from the topmost atomic layers and the deconvolution affects little at the topmost surface. However, in S3 with higher doping density, *E*_s_ shifts away from *E*_0,_ indicating the inaccuracy of linear potential approximation. For S1 and S2, the space charge region width is dozens of nanometer, which is much larger than the  detection depth, so we adopt the linear potential approximation at the surface (details are shown in the supplementary information). Owing to the high doping level in S3, the width of the space charge region is reduced to be comparable with the photoelectron depth, thus the realistic quadratic depletion approximation should be more suitable.

**Figure 5:**
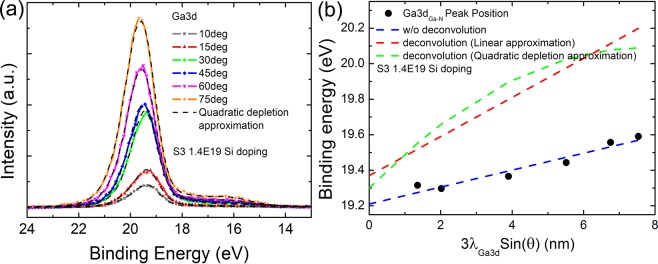
Quadratic depletion approximation correction in highly Si doped GaN films (S3). (**a**) The experimentally observed Ga 3d spectra of S3 at different emission angles are shown by solid symbol- lines. The black dashed lines are the fittings using the quadratic depletion approximation by Eqs (2, 3). (**b**) The binding energy of Ga 3d _(Ga-N)_ peak as a function of detection depth with carrier concentration of 1.4 × 10^19^ cm^−3^ (S3). The blue dashed lines are the linear fitting of the measured binding energy peak without deconvolution. The red dashed line performed the deconvolution of the spectra by linear approximation and the green dashed lines show the deconvolution of the spectra by quadratic depletion approximation.

To gain further insights in highly doped GaN sample, the quadratic depletion approximation correction was considered. In the depletion approximation, *ψ*(z) was treated as $\psi (z)\approx {\psi }_{s}{(1-\sqrt{\frac{q{N}_{d}}{2{\varepsilon }_{GaN}{\psi }_{s}}}z)}^{2}$, where *ψ*_s_ is the surface potential at *z* = 0, *q* is the electronic charge, *N*_d_ is the doping density, and *ε*_GaN_ is the dielectric constant of GaN^25^. As shown in Fig. 5(a), the experimentally observed Ga 3d spectra measured at different emission angles were quantitatively in agreement with the quadratic depletion approximation fitting (black dashed lines). The extracted binding energy at topmost surface, *E*_0_, is 19.30 eV. The actual core level binding energy as a function of detection depth after considering the effect of quadratic depletion approximation by Eqs (2, 3) is shown by the green dashed line in Fig. 5(b). Compared with the linear potential approximation (red dashed line in Fig. 5(b)), the deviation between *E*_0_ and *E*_s_ becomes smaller. The quadratic depletion approximation is closer to the experimental results in surface layers.

To sum up, for the moderately doped GaN, due to the larger depletion width, the effective electron surface potential can be reflected by the simply linear approximation, while in highly doped GaN, the quadratic depletion approximation is more applicable. Moreover, by calculating the surface band bending, according to Eq. (1), the surface band bending for three different doping densities after deconvolution correction are calculated to be 1.53 eV, 1.72 eV, and 1.73 eV for S1, S2 and S3 respectively.

## Conclusion

In summary, we performed a systematic study on the surface band bending of Ga-polar n-GaN with different Si doping density via ADXPS. The binding energies for Ga 3d and N 1 s in GaN films increase with increasing the detection depth, implying an upward surface band bending. Considering the effect of integration caused by internal electric field in band bending samples, we corrected the core level binding energies by subtracting the integrated effect and correctly determined the value of surface band bending. Our study confirms the major contributions come from the surface layers. For moderately doped GaN, the effective electron surface potential can be represented by a simply linear approximation. For highly doped GaN, where the photoelectron emission depth is comparable to the width of the space charge region, a quadratic depletion approximation should be adapt to simulate the internal potential distribution with band bending effect.

## Methods

### Sample Preparation

Three different Si doped Ga-polar n-GaN films with thickness of 1μm were grown on 2-inch p-type Si (111) substrates with undoped GaN buffer layers of 1.5 μm in between by metal organic chemical vapor deposition. The carrier concentrations of the three samples are in the range of (8.5–9.5)* × *10^17^ cm^−3^ for Sample 1, 4 × 10^18^ cm^−3^ for Sample 2, and 1.4 × 10^19^ cm^−3^ for Sample 3, as characterized by using Hall measurement and verified by secondary ion mass spectroscopy (SIMS).

### ADXPS characterization

ADXPS was carried out by using a PHI 5000 Versaprobe II system equipped with a monochromatic Al Kα (1486.6 eV) X-ray source, to obtain the core level and valance band structure spectra of all samples. The core level spectra were subtracted by a Shirley-type background and fitted using combined Gaussian and Lorentzian line shapes. The valance band maxima were determined by extrapolating a linear fit of the leading edge of the valance band photoemission to the baseline. The binding energy calibration was performed by using gold (Au), silver (Ag), and copper (Cu) standard samples by setting Au 4f_7/2_, Ag 3d_5/2_, Cu 2p_3/2_ peaks at binding energies of 83.96 ± 0.1 eV, 368.21 ± 0.1 eV and 932.62 ± 0.1 eV, respectively. The XPS spectra were performed at different emission angles *θ* from 10° to 85°, with respect to the sample surface, ranging. The Fermi edge was calibrated using a pure and *in-situ* cleaned silver (Ag) standard sample and setting the binding energy at 0.00 eV. To further calibrate the charging effect, the spectra were referenced to the peak position of C1s core levels to 284.8 eV for each sample.

### TOF-SIMS characterization

The concentration of doped Si in n-GaN were verified by a TOF-SIMS depth profile technique, using a Bi+ ion beam with energy of 30 keV and pulsed current of 3.5 pA for analysis. Depth sputtering was performed using a Cs+ beam of 2 keV and 75 nA to produce a crater of 200 × 200 μm. The analysis area was 50 × 50 μm in the center of the crater. Si- ions were collected at negative ion detection mode.

## Supplementary information

Supplementary Information
