# Supplementary material for: Precise determination of surface band bending in Ga-polar n-GaN films by angular dependent X-Ray photoemission spectroscopy
Source: Sci Rep. 2019 Nov 18;9:16969. doi: 10.1038/s41598-019-53236-9 (PMC6861320; doi:10.1038/s41598-019-53236-9)
Supplement: Supplementary file 1 — Supplementary Information [file 41598_2019_53236_MOESM1_ESM.docx]

**Supplementary Information**

**Precise determination of surface band bending in Ga-polar n-GaN films by angular dependent X-Ray photoemission spectroscopy**

Yanfei Zhao^1,*^, Hongwei Gao^2^, Rong Huang^1^, Zengli Huang^1^, Fangsen Li^1^, Jiagui Feng^1^, Qian Sun^2^, An Dingsun^1,*^ & Hui Yang^1, 2^

^1^ Vacuum Interconnected Nanotech Workstation (Nano-X), Suzhou Institute of Nano-Tech and
Nano-Bionics (SINANO), Chinese Academy of Sciences (CAS), Suzhou 215123, China
^2^ Key Laboratory of Nanodevices and Applications, Chinese Academy of Sciences (CAS), Suzhou 215123, China

Correspondence and requests for materials should be addressed to:

Y. Z. (yfzhao2015@sinano.ac.cn); A. D.(adingsun2014@sinano.ac.cn)

**1. Quadratic depletion approximation correction for S1 and S2**

The quadratic depletion approximation correction was considered in S1 and S2. In Fig. S1(a) and S1(c), the black dashed lines are displayed the quadratic depletion approximation fitting of the Ga 3d spectra measured at different emission angles for S1 and S2 respectively. Extracting from the fitting, the depletion layer width can be calculated by $w=\sqrt{\frac{2\varepsilon_{GaN}\psi_{s}}{qN_{d}}}$. For S1 and S2, the depletion layer width was calculated to be 73.3 nm and 16.8 nm respectively, both of which are much larger than the photoelectron detection depth 3*λ*_Ga3d_=7.8 nm.

The actual core level binding energy as a function of detection depth after considering the effect of quadratic depletion approximation by Equation (2, 3) in the main text is shown by the green dashed line in Fig. S1(b) and S1(d). Compared with the linear potential approximation (red dashed line in Fig. S1(b) and S1(d)), the deviation between *E*_0_ and *E*_s_ is small. For S1 and S2, the quadratic depletion approximation is almost same as the linear potential approximation within the photoelectron detection region. In other words, for the moderately doped GaN (S1 and S2), when the depletion width is much larger than the photoelectron detection depth, the quadratic depletion approximation can be instead by the simply linear approximation.


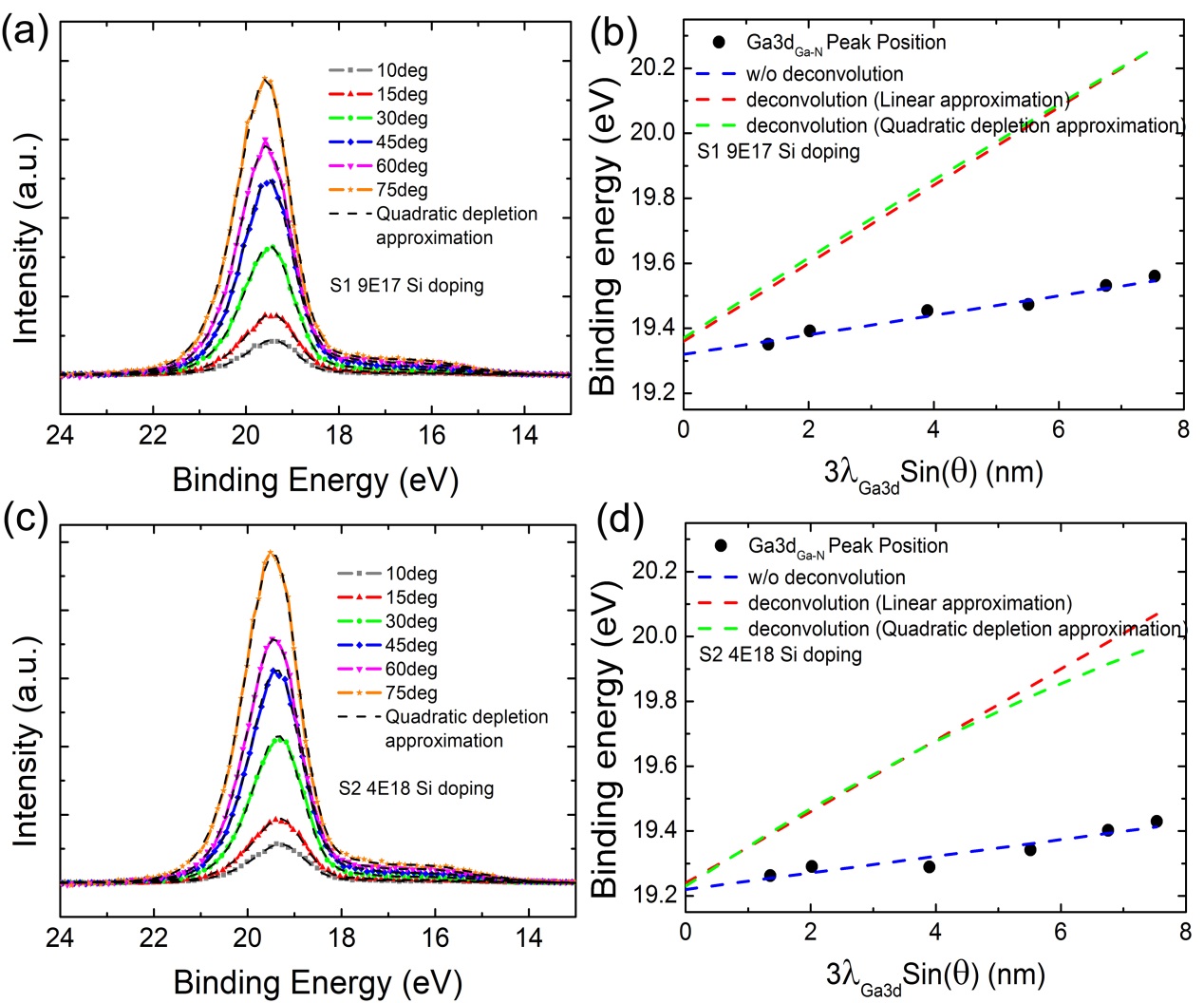


Figure S1 Quadratic depletion approximation correction in S1 and S2. (a) and (c) The experimentally observed Ga 3d spectra of S1 and S2 at different emission angles are shown by solid lines. The black dashed lines are the fittings using the quadratic depletion approximation by Equation (2, 3) in the main text. (b) and (d) The binding energy of Ga 3d _(Ga-N)_ peak as a function of detection depth for S1 and S2, respectively. The blue dashed lines are the linear fitting of the measured binding energy peak without deconvolution. The red dashed line performed the deconvolution of the spectra by linear approximation and the green dashed lines show the deconvolution of the spectra by quadratic depletion approximation.

**2. Quadratic depletion approximation correction for S3**

Extracting from the fitting, the depletion layer width can be calculated by $w=\sqrt{\frac{2\varepsilon_{GaN}\psi_{s}}{qN_{d}}}$. For S3, the depletion layer width was calculated to be 7.7 nm, which is comparable with the photoelectron detection depth 3*λ*_Ga3d_=7.8 nm. As shown in Fig. 5(b) in the main text, the quadratic depletion approximation is obvious different from linear potential approximation within the photoelectron detection region. Thus, in highly doped GaN (S3), the quadratic depletion approximation is more applicable.
